# Outcomes of thromboprophylaxis with enoxaparin vs. unfractionated heparin in medical inpatients

**DOI:** 10.1186/1477-9560-4-17

**Published:** 2006-09-27

**Authors:** Lisa J McGarry, Michael E Stokes, David Thompson

**Affiliations:** 1 Health Economics & Outcomes Research, i3 Innovus, 10 Cabot Rd., Suite 304, Medford, MA 02155-5173, USA; 2 Center for Health Economics & Policy, United BioSource Corporation, 101 Station Landing, Medford, MA 02155, USA; 3 Global Health Economics, i3 Innovus, 10 Cabot Rd., Suite 304, Medford, MA 02155-5173, USA

## Abstract

**Background:**

Clinical trials have shown low-molecular weight heparin (LMWH) to be at least as safe and efficacious as unfractionated heparin (UFH) for preventing venous thromboembolism (VTE) in acutely-ill medical inpatients.

**Objective:**

To compare clinical and economic outcomes among acutely-ill medical inpatients receiving the LMWH enoxaparin versus UFH prophylaxis in clinical practice.

**Methods:**

Using a large, multi-hospital, US database, we identified persons aged ≥40 years hospitalized for ≥6 days for an acute medical condition (including circulatory disorders, respiratory disorders, infectious diseases, or neoplasms) from Q4 1999 to Q1 2002. From these patients, those who received thromboprophylaxis with either enoxaparin or UFH were identified. Surgical patients and those requiring or ineligible for anticoagulation were excluded. We compared the incidence of deep-vein thrombosis (DVT), pulmonary embolism (PE), and all VTE (i.e., DVT and/or PE). Secondary outcomes were occurrence of side-effects, length of hospital stay and total costs. RESULTS: 479 patients received enoxaparin prophylaxis and 2,837 received UFH. The incidence of VTE was 1.7% with enoxaparin prophylaxis versus 6.3% with UFH (RR = 0.26; p < 0.001). Occurrence of side effects, length of stay (10.00 days with enoxaparin vs. 10.26 days with UFH; p = 0.348) and total costs ($18,777 vs. $17,602; p = 0.463) were similar in the 2 groups.

**Conclusion:**

We observed a 74% lower risk of VTE among patients receiving enoxaparin prophylaxis versus UFH prophylaxis. There was no significant difference in side effects or economic outcomes. These results provide evidence that the LMWH enoxaparin is more effective than UFH in reducing the risk of VTE in current clinical practice.

## Background

Acutely-ill medical inpatients – such as those hospitalized for congestive heart failure (CHF), chronic obstructive pulmonary disease (COPD), acute infections, or cancer – often have prolonged hospital stays with periods of immobility that place them at increased risk of venous thromboembolism (VTE) [1]. Because these patients frequently have additional risk factors (e.g., history of VTE, advanced age, obesity, varicose veins, estrogen use), the potential benefits of thromboprophylaxis in this population are substantial [1]. The two most commonly-used methods of thromboprophylaxis in acutely-ill medical patients are low-molecular weight heparin (LMWH) and unfractionated heparin (UFH) [2]. Clinical trials conducted among medical inpatients have shown thromboprophylaxis with LMWH to be at least as safe and efficacious as UFH in this population^, ^and LMWH may offer clinical advantages over UFH, including longer duration of action, more predictable response, and once-daily subcutaneous administration [3-7]. However, the outcomes of using these prophylaxis methods in the real-world clinical setting have not been examined. The purpose of this study was to compare the clinical and economic benefit of these two methods of thromboprophylaxis using data from real-world clinical practice. The study time period was from the Q4 1999 through the Q1 2002. The study focuses on the LMWH enoxaparin, which is the most widely used LMWH in the US and the most frequently studied in this indication.

## Methods

### Data source

Data are from the Cerner HealthFacts Database, a data warehouse containing hospital records for approximately 2.8 million patients from acute-care institutions throughout the U.S. Patient records include principal and secondary diagnoses (in ICD-9-CM format); inpatient procedures (in ICD-9-CM format) and procedure date; and drugs dispensed (in National Drug Code format) with dispensing date, dosage, and frequency of administration. Patient demographic information (age, sex, race) and descriptive hospital information (geographic region, number of beds, teaching vs. nonteaching) are provided. Dates of admission and discharge, discharge status, and total billed charges are reported for each inpatient stay.

Patient records in this database have been de-identified in compliance with the Health Insurance Portability and Accountability Act of 1996 (HIPAA) Privacy Rule, and records relating to a common hospital discharge are linked using a non-personal identifier assigned by the data vendor [8]. The dataset obtained for this study encompassed Q4 1999 through the Q1 2002.

### Sample selection

To select patients at high risk of thromboembolism, the dataset included only those aged ≥ 40 years at admission with an inpatient stay ≥ 6 days. We focused on patients with serious medical conditions by excluding patients undergoing surgery (see additional file 1) within two days of admission. We then classified patients based on the ICD-9-CM code (inclusive of all 4^th ^& 5^th ^digit classifications) recorded as their principal diagnosis and selected only those patients with principal diagnoses of: (1) respiratory disorders (ICD-9-CM 460–519, 748, 786, 996.84, 997.3); (2)circulatory disorders (390–398, 410–459, 997.1, 997.2); (3)infectious diseases (001–139, 680–686, 730, 996.6, 997.62, 998.3, 998.5, 999.3); and (4)neoplasms (140–239). We excluded as medically ineligible for thromboprophylaxis, patients with a diagnosis of active peptic ulcer (ICD-9-CM 530.2, 531.0–531.3, 532.0–532.3, 533.0–533.3, 534.0–534.3), malignant hypertension, including renal disease with or without renal failure (401.0, 402.0, 403.0, 404.0, 405.0), blood diseases (280–289), or HIV infection (V08, 042, 079.53), as well as those undergoing intubation of the gastrointestinal or respiratory tracts (96.0) within the first two days of admission. Pregnant women were excluded based on a diagnosis of pregnancy (630–677) or evidence of obstetrical procedures (72–75). The specific disorders corresponding to each ICD-9-CM code for inclusion and exclusion criteria are listed in additional file 1.

We selected patients receiving prophylaxis with enoxaparin or UFH by calculating the daily dosage of anticoagulants dispensed within the first two days of admission. Patients receiving an enoxaparin dosage of 30–60 mg/day were deemed to be receiving enoxaparin thromboprophylaxis and those receiving a UFH dosage between 5,000 and 15,000 IU/day to be receiving UFH thromboprophylaxis. Those who received any combination of enoxaparin and IV UFH, or received another anticoagulant in addition to enoxaparin or UFH (including dalteparin, tinzaparin, ardeparin, nadroparin, or warfarin), during the first two days of admission were excluded from the analysis. In addition, patients who received high-dose enoxaparin (>100 mg/day) or UFH (>25,000 IU/day) within two days of admission were assumed to be receiving treatment for VTE or other conditions present at admission, and were excluded. Because enoxaparin dosages of >60 to <100 mg/day and UFH dosages of >15,000-<25,000 IU/day could not be classified definitively as prophylaxis vs. treatment, patients receiving dosages in these ranges within two days of admission also were excluded. Patients were allowed to receive up to 48 hours of low-dose therapy with a second anticoagulant at any point after their first two days in hospital; those receiving >48 hours were excluded unless this therapy was subsequent to a diagnosis of VTE. Because patients may receive anticoagulant treatment for non-thrombotic disorders, we excluded those who received high-dose anticoagulant at any point during their hospital stay and had a diagnosis of myocardial infarction (ICD-9-CM 410), cardiac dysrhythmia (427), angina (413), or valve disorders (394–397, 424). Finally, to ensure that we had a complete record for each hospitalization, we excluded patients missing discharge diagnoses and/or billed charges, and those transferred from or discharged to another acute-care facility. Sample selection and exclusion criteria were specified a priori, except those related to drug dosing, which were finalized after examination of the data.

### Study measures

The primary outcome measure was the occurrence of VTE (deep-vein thrombosis [DVT] and/or pulmonary embolism [PE]) during the hospitalization. A diagnosis of DVT was identified by either: (1) an ICD-9-CM diagnosis of 451–453 (inclusive of all 4^th ^and 5^th ^digit classifications); or (2) receipt of a therapeutic dosage of either enoxaparin (i.e., >100 mg/day) or heparin (>25,000 IU/day) at any time after the first two inpatient days. A diagnosis of PE was identified by ICD-9-CM code 415.1. Secondary outcome measures were the occurrence of major bleeds (ICD-9-CM 286.5, 430–432, 459.0, 578, 786.3) and thrombocytopenia (287.4–287.5), as well as death in hospital, length of hospital stay, and estimated inpatient costs. Specific diagnoses corresponding to each ICD-9-CM code are detailed in additional file 1. Because the ICD-9-CM diagnosis codes used to identify bleeding and thrombocytopenia are not associated with a date of diagnosis, we were not able to determine if these outcomes occurred after receipt of the study drug, therefore all occurrences of these outcomes were considered. Similarly, because the cause of death was not available, all deaths in hospital were considered.

### Data analysis

Descriptive characteristics of the sample were examined and risk factors for VTE identified by ICD-9-CM diagnosis code, including CHF (428.0), COPD (490–492, 496), fracture of pelvis, femur or tibia (808, 820–821), inflammatory bowel disease (558.9), and nephrotic syndrome (581). Drug records were inspected to identify patients receiving oral estrogen. Dosing for each method of prophylaxis also was noted. Costs of hospitalization were estimated from billed charges using cost-to-charge ratios from the Centers for Medicare & Medicaid Services (CMS) Prospective Payment System (PPS) [9]. Because information necessary to link the discharging institutions to their corresponding CMS records was not available, we matched records to average cost-to-charge ratios by region, hospital size (i.e., bed count category), and teaching versus non-teaching status, and used these ratios to estimate costs. Categorical outcomes were compared between patients receiving prophylaxis with enoxaparin versus UFH using risk-ratios and two-sided Χ^2 ^statistics, or the Fischer's exact test where expected cell frequencies were <5; differences in continuous measures were compared using Student's *t*-test.

Patients in the database were not randomly assigned to prophylaxis; therefore we used the propensity score method to control for confounding [10]. To assign a propensity score to each patient, a logistic regression model predicting receipt of enoxaparin prophylaxis as a binary outcome from patient and hospital characteristics was estimated from the data. The propensity scores range between zero and one, representing each patient's predicted probability of receiving enoxaparin (versus UFH) prophylaxis conditional on his or her baseline covariates. Various predictive models were evaluated by comparing the difference in -2 log likelihoods to a Χ^2 ^distribution with the appropriate degrees of freedom and rejecting non-significant covariates at α = 0.10.

The propensity score generated for each patient from the final model was used to perform a matched analysis in which patients were divided into five strata (i.e., quintiles) based on the propensity score distribution in the overall sample. For categorical measures, stratum-specific risk ratios were calculated among the patients in each quintile. These risk ratios were combined using the Mantel-Haenszel method, and compared using the Mantel-Haenszel Χ^2 ^test; homogeneity of risk-ratios across the strata was evaluated using the Breslow-Day test. For continuous measures, means calculated within each quintile were averaged across the quintiles and differences between treatment groups compared using the F-statistic. Homogeneity was tested using an interaction term for prophylaxis group and stratum.

Stratified analyses by dose and duration of prophylaxis were conducted to examine the effect of treatment regimen on outcomes. Lastly, sensitivity analyses were performed to examine the effect of possible misclassification of patients by drug exposure or outcome on our findings. Except where noted, results were considered to be statistically significant at α = 0.05.

## Results

### Patient characteristics

**Figure 1:**
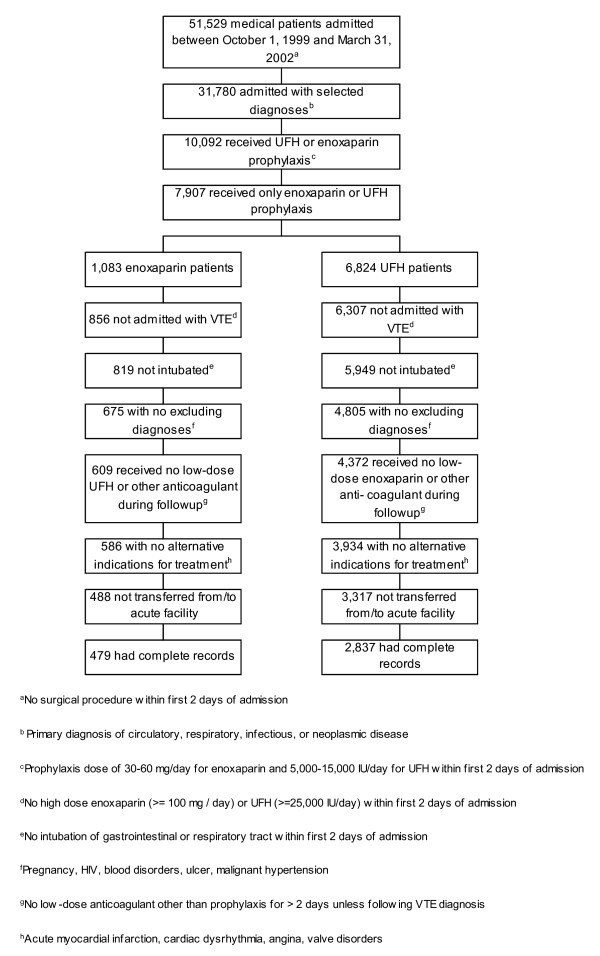
Patients included in the analysis and reason for exclusion.

We identified 479 acutely-ill medical inpatients receiving enoxaparin prophylaxis and 2,837 receiving UFH prophylaxis who conformed to study inclusion and exclusion criteria [Figure 1]. The median daily dose of enoxaparin received by the enoxaparin prophylaxis cohort was 60 mg/day. Sixty-one percent of enoxaparin patients received this dose, 36% received 40 mg/day and the remainder received either 30 or 50 mg/day. The median daily dose of UFH, received by 78% of patients in the UFH prophylaxis cohort, was 10,000 IU/day; 14% received a lower and 8% a higher dose.

Enoxaparin patients were older than UFH patients and were more likely to be female and white (Table 1). The distribution of diagnoses in the two groups was roughly the same; however, patients with circulatory disorders comprised a larger proportion of the patients in the UFH cohort, while those with acute infections were a larger proportion of the enoxaparin patients. Risk factors for VTE were similar in the two groups, with the exception of CHF and fractures of the lower limb, which were significantly more common among enoxaparin patients than UFH patients. The patient sample was weighted toward the northeastern region, and enoxaparin prophylaxis patients were more likely to have been admitted to a hospital in the northeast and to moderate-sized, non-teaching hospitals than UFH patients.

**Table 1:** Characteristics of medical inpatients receiving enoxaparin versus UFH prophylaxis

	Prophylaxis Received		
		
Characteristic	Enoxaparin	UFH	p-value
N	479	2,837	
Age (Mean [Range])	75.6 (40–98)	71.8 (40–101)	<0.001
Age group [n (%)]:			
40–49 years	30 (6.3)	185 (6.5)	<0.001
50–59 years	39 (8.1)	414 (14.6)	
60–69 years	67 (14)	527 (18.6)	
70–79 years	131 (27.3)	850 (30.0)	
80–89 years	165 (34.4)	687 (24.2)	
90+ years	47 (9.8)	174 (6.1)	
Female [n (%)]	285 (59.5)	1,506 (53.1)	0.009
White [n (%)]	449 (93.7)	2,302 (81.1)	<0.001
Principal diagnosis [n (%)]:			
Circulatory	215 (44.9)	1,349 (47.6)	0.062
Respiratory	150 (31.3)	926 (32.6)	
Acute Infection	70 (14.6)	296 (10.4)	
Neoplasm	44 (9.2)	266 (9.4)	
Risk factors for VTE [n (%)]:			
CHF	194 (40.5)	810 (28.6)	<0.001
COPD	156 (32.6)	826 (29.1)	0.126
Receipt of HRT	19 (4.0)	127 (4.5)	0.615
Obesity (coded as medical condition)	21 (4.4)	123 (4.3)	0.962
Inflammatory bowel disease	3 (0.6)	6 (0.2)	0.129
Fracture of lower limb	3 (0.6)	3 (0.1)	0.043
Nephrotic syndrome	1 (0.2)	5 (0.2)	1.000
Geographic region of hospital [n (%)]:			
Northeast	374 (78.1)	1,227 (43.2)	<0.001
Midwest	21 (4.4)	681 (24.0)	
South	79 (16.5)	916 (32.3)	
West	5 (1.0)	13 (0.5)	
Hospital size [n (%)]:			
< 200 beds	30 (6.3)	214 (7.5)	<0.001
200–499 beds	273 (57.0)	864 (30.5)	
500+ beds	176 (36.7)	1,759 (62.0)	
Teaching hospital [n (%)]	260 (54.3)	2,235 (78.8)	<0.001

VTE = venous thromboembolism, CHF = congestive heart failure, COPD = chronic obstructive pulmonary disease, HRT = hormone replacement therapy

### Outcomes and costs

The overall incidence of DVT among study subjects was 5.2%, and the incidence of all VTE (DVT and/or PE) was 5.7%. The risks of PE (p = 0.010), and all VTE (p < 0.001) over the course of hospitalization were significantly lower in the group receiving enoxaparin prophylaxis than among those receiving UFH prophylaxis (Table 2). There was no significant difference in the risks of major bleeds (p = 0.966) and death in hospital (p = 0.843) between enoxaparin versus UFH patients, and no thrombocytopenia was recorded among the patients in either group. Similarly, there was little difference in hospital length of stay (p = 0.348) and costs of hospitalization (p = 0.463) between patients receiving enoxaparin versus UFH prophylaxis (Table 3).

**Table 2:** Incidence of venous thromboembolism and adverse events among medical inpatients receiving enoxaparin versus UFH prophylaxis

	Prophylaxis Received [N(%)]		
		
Outcome	Enoxaparin (N = 479)	UFH (N = 2,837)	RR (95% CI)
DVT	8 (1.7)	163 (5.8)	0.29 (0.14, 0.59)
PE	0 (0.0)	32 (1.1)	0
VTE (DVT and/or PE)	8 (1.7)	180 (6.3)	0.26 (0.13,0.53)
Adverse Events:			
HIT	0 (0.0)	0 (0.0)	--
Major Bleed	12 (2.5)	72 (2.5)	0.99 (0.54,1.80)
Death	25 (5.2)	142 (5.0)	1.04 (0.69, 1.58)

DVT = deep-vein thrombosis; PE = pulmonary embolism; VTE = venous thromboembolism; HIT = heparin-induced thrombocytopenia

**Table 3:** Length of stay in hospital and estimated costs among medical inpatients receiving enoxaparin versus UFH prophylaxis

	Prophylaxis Received		
		
Outcome (Mean)	Enoxaparin	UFH	Difference (95% CI)
Length of hospital stay (days)	10.00	10.26	-0.26 (-0.99, 0.46)
Total costs	$18,777	$17,602	$1,174 (-2,365, 4,714)

### Propensity score analysis

The variables chosen as important independent predictors of receipt of enoxaparin versus UFH were sex, race (white vs. nonwhite), age group (in 10-year increments), diagnosis group, presence of CHF, interaction of CHF and diagnosis group, fracture of lower limb, hospital region, hospital size (bed count category) and teaching status. Covariates were, in general, balanced within the strata.

The adjusted relative risks of DVT (0.25; 95% CI: 0.12, 0.51) and VTE (0.24; 95% CI: 0.12, 0.50) for patients receiving enoxaparin versus UFH prophylaxis were similar to, and somewhat more favorable for enoxaparin, than the unadjusted estimates. The adjusted estimates of the relative risks of major bleed (1.56; 95% CI 0.83, 2.91) and death (0.85; 95% CI 0.55, 1.31), while further from the null than the unadjusted estimates, indicate no significant difference between patients receiving enoxaparin versus UFH prophylaxis. We similarly found no significant difference between the two groups with regard to length of inpatient stay (-0.21; 95% CI -0.99, 0.58) and total costs ($1,249; 95% CI -2,510, 5,008).

### Stratified analyses

To explore the effect of dosing of prophylaxis on occurrence of VTE, we classified patients receiving enoxaparin into two groups: those receiving a dosage of 30–40 mg and those receiving 50–60 mg, and classified patients receiving UFH into three groups: those receiving 5,000-<10,000 IU, those receiving 10,000 IU, and those receiving >10,000–15,000 IU and examined outcomes in these groups. Using the F-test for trend, we found that the risk of VTE decreased significantly for UFH patients as the dosage of UFH increased, but observed a significant increase in the risk of VTE among enoxaparin patients at higher dosages (Table 4).

**Table 4:** Incidence of venous thromboembolism among medical inpatients receiving enoxaparin versus UFH prophylaxis by dosage and duration of prophylaxis received

			Outcome (N [%])		
			
Prophylaxis Received/Dosage or Days	N	% of Patients	DVT	PE	VTE (DVT and/or PE)
***Dosage:***					
Enoxaparin					
30–40 mg	186	38.8%	**0 (0.0)**	0 (0.0)	**0 (0.0)**
50–60 mg	293	61.2%	**8 (2.7)**	0 (0.0)	**8 (2.7)**
UFH					
5,000-<10,000 IU	414	14.5%	**47 (11.4)**	8 (1.93)	**52 (12.6)**
10,000 IU	2,221	78.0%	**109 (4.9)**	23 (1.04)	**121 (5.4)**
>10,000–15,000 IU	214	7.5%	**7 (3.3)**	1 (0.5)	**7 (3.3)**
					
***Prophylaxis days:***					
Enoxaparin					
1–2 days	74	15.4%	**4 (5.4)**	0 (0.0)	**4 (5.4)**
3–4 days	36	7.5%	**2 (5.6)**	0 (0.0)	**2 (5.6)**
5–6 days	123	25.7%	**2 (1.6)**	0 (0.0)	**2 (1.6)**
> 6 days	246	51.4%	**0 (0.0)**	0 (0.0)	**0 (0.0)**
UFH					
1–2 days	831	29.3%	**109 (13.1)**	**22 (2.7)**	**120 (14.4)**
3–4 days	227	8.0%	**21 (9.25)**	**2 (0.9)**	**23 (10.1)**
5–6 days	608	21.4%	**15 (2.5)**	**4 (0.7)**	**15 (2.5)**
> 6 days	1,171	41.3%	**18 (1.5)**	**4 (0.3)**	**22 (1.9)**

Bold indicates p for trend <0.05

To examine the effect of duration of prophylaxis on outcome, we next stratified patients in both treatment groups into 4 groups: those receiving 1–2 days of prophylaxis; 3–4 days; 5–6 days and > 6 days. Because we required patients in our study sample to have been hospitalized for a minimum of 6 days, all patients had the opportunity to receive up to 6 days of prophylaxis. Patients receiving with UFH tended to have fewer days of prophylaxis than those receiving enoxaparin. In both groups, the risk of VTE decreased significantly with increased duration of prophylaxis (Table 4).

### Sensitivity analyses

To examine the importance of using drug records to identify patients with VTE, we reanalyzed the data entirely disregarding drug treatment as a marker for VTE and classifying only those patients with a recorded ICD-9-CM diagnosis code for DVT or PE as having VTE. The risk-ratio for VTE for patients receiving enoxaparin versus UFH prophylaxis using this narrower definition was 0.36 (p = 0.021). Next we explored the effect of possible misclassification of patients by receipt of enoxaparin and UFH prophylaxis, by restricting patients to only those receiving the recommended prophylaxis dosage of 40 mg/day of enoxaparin (n = 173) or 15,000 IU/day UFH (n = 211). In this subgroup, there were no cases of VTE among those receiving enoxaparin prophylaxis versus a risk of 3.3% for those receiving UFH prophylaxis (p = 0.018).

## Discussion

Our comparison of enoxaparin versus UFH in real-world clinical practice yielded results more favorable for enoxaparin than those from clinical trials, which have concluded equivalence between these two prophylaxis methods [3-7]. This difference in outcome may be explained in part by differences in the drug dosing used in the trials versus those we observed in clinical practice. The two trials in which enoxaparin was nominally (but not significantly) less efficacious than UFH administered dosages of 20 and 36 mg/day of enoxaparin and 15,000 IU/day of UFH, in contrast to the median dosages of 60 mg/day and 10,000 IU/day, that we observed in practice [4,5]. Two other trials that found enoxaparin to be more efficacious (but not significantly) than UFH, used dosages of 40 mg/day for enoxaparin and 15,000 IU/day for UFH, which are consistent with current recommendations [1,6,7]. The reasons for the use of higher-than-recommended dosages of enoxaparin and lower-than-recommended dosages of UFH that we observed in real-world practice are unknown, but may reflect differences in ease of administration and monitoring, as well as perceptions about the relative safety of the two prophylaxis methods.

When we stratified patients by dosage of anticoagulant received we found a significant (p < 0.05) trend of fewer VTEs with higher dosing among UFH patients, but the opposite trend among enoxaparin patients. The reason for this seemingly contradictory finding is unclear and may suggest that some of the patients receiving higher dosages of enoxaparin were receiving treatment rather than prophylaxis for VTE. This is consistent with our finding that the 90 patients receiving a dosage of UFH >15,000 IU to 20,00 IU not included in our analysis had a higher rate of VTE (12%) than the patients receiving dosages between 5,000–15,000 IU included in the study. In any case, the results of the stratified analyses suggest that some cases of VTE among UFH patients may be related to inadequate UFH dosing. However in sensitivity analyses that compared patients receiving the recommended dosages of 40 mg/day of enoxaparin and 15,000 IU/day of UFH, the occurrence of VTE among enoxaparin patients remained significantly lower than that among UFH patients.

We similarly stratified patients by duration of prophylaxis and found a significant trend of fewer VTEs with longer duration of thromboprophylaxis. Because patients receiving UFH prophylaxis tended to have fewer days of prophylaxis than those receiving enoxaparin, some cases of VTE among UFH patients may be related to shorter duration or prophylaxis. We note, however, that the occurrence of VTE was lower among enoxaparin versus UFH patients at all levels of treatment duration.

Despite the significantly lower overall risk of VTE among enoxaparin versus UFH patients, we observed no economic benefit of thromboprophylaxis with enoxaparin versus UFH in terms of reduction in either length of hospital stay or total inpatient costs. When pooled across the two treatment groups, patients with VTE did have significantly longer inpatient stays than those without (14.8 days vs. 9.9 days; p = 0.001) and correspondingly higher inpatient costs ($26,605 vs. $17,241; p = 0.003); however, these differences did not translate into overall lower costs for enoxaparin patients. This lack of apparent economic benefit for enoxaparin prophylaxis versus UFH prophylaxis may be due to the relatively small number of patients experiencing VTE, our inability to isolate costs directly related to VTE from the total costs of hospitalization, and the high degree of variability in both length of stay (ranging from 6 to 54 days) and inpatient costs (from $3,000 to $384,000) among the patients in this sample.

Our study is subject to the limitations inherent in the use of retrospective administrative data. First, we identified and classified study subjects based on drug records and ICD-9-CM coding of diagnoses and procedures taken from the inpatient record. If these records were inaccurate or incomplete, subjects may have been misidentified as eligible, or misclassified by diagnosis or receipt of prophylaxis. Similarly, the incidence of VTE was derived from information recorded by the treating institutions; therefore, the validity of our findings depends on the accuracy of their record keeping and on our interpretation of the data. Drug records were used both to classify patients receiving prophylaxis, and to identify patients experiencing VTE, and assumptions regarding treatment patterns and dosing were necessary. Sensitivity analyses demonstrated, however, that our results were not highly dependent on these assumptions, and our conclusions were similar when we used narrower definitions of prophylaxis and treatment.

We further note that because VTE was identified from administrative records, it was not possible to distinguish between symptomatic and asymptomatic VTEs. Attempts to identify patients receiving diagnostic testing for VTE suggested low levels of reporting for these tests, and available data did not allow us to determine the outcome of the test except as inferred from treatment received. In addition, because of the limited information available for the bleeds identified from ICD-9 diagnostic coding, we cannot be certain that these outcomes were caused by administration of either study drug.

Because patients were not randomly assigned to enoxaparin versus UFH prophylaxis, underlying differences in the two groups may have influenced both the selection of thromboprophylaxis and the occurrence of VTE. We note, however, that for our results to have been produced solely by "confounding by indication", treating physicians would have to systematically administer enoxaparin prophylaxis to patients at lower risk of VTE than those receiving UFH prophylaxis. In fact, the enoxaparin patients in our sample had nominally higher frequencies of nearly every risk factor for VTE examined. Moreover, using propensity score techniques to control for confounding, we found that enoxaparin patients had lower risks of VTE than UFH patients across all strata. Nevertheless, the extent of uncontrolled confounding in this study remains unknown.

Finally, in selecting patients for this study, we excluded groups such as patients aged <40 years, pregnant women, and patients with certain comorbid conditions; therefore, extrapolation of our finding to these populations may not be appropriate.

## Summary

Using hospital administrative data, we observed a 74% lower risk of VTE among acutely-ill medical inpatients receiving enoxaparin prophylaxis versus UFH prophylaxis, but found no difference in occurrence of side-effects, death in hospital, length of hospital stay, or inpatient costs. We conclude that enoxaparin is more effective than UFH in reducing the risk of VTE in acutely-ill medical patients in current clinical practice.

## Conflict of interest

Funding for this research was provided by Aventis Pharmaceuticals, Bridgewater, NJ, which manufactures enoxaparin (Lovenox^®^). The authors are paid consultants to Aventis.

## Supplementary Material

Additional File 1 Appendix. Included diseases and corresponding ICD-9 CM codes. Click here for file
